# Selection of Reference Genes for qRT-PCR Analysis in *Lentinula edodes* after Hot-Air Drying

**DOI:** 10.3390/molecules24010136

**Published:** 2018-12-31

**Authors:** Shuangshuang Gao, Gangzheng Wang, Zhicheng Huang, Xiaoyu Lei, Yinbing Bian, Ying Liu, Wen Huang

**Affiliations:** 1 College of Food Science and Technology, Huazhong Agricultural University, Wuhan, Hubei 430070, China; 13720161459@163.com (S.G.); HuangZhiCheng1210@163.com (Z.H.); xiaoyulei1988@126.com (X.L.); 2 Institute of Applied Mycology, Plant Science and Technology College, Huazhong Agricultural University, Wuhan, Hubei 430070, China; wgzhau@163.com (G.W.); bianyinbing@mail.hzau.edu.cn (Y.B.)

**Keywords:** *Lentinula edodes*, hot-air drying, volatile sulfur compounds, qRT-PCR, reference genes

## Abstract

Volatile sulfur compounds gradually develop in *Lentinula edodes* after hot-air drying, and many genes are involved in the generation of these sulfur compounds. The expression stability of reference genes may vary in a particular experimental treatment when analyzing their expressions by quantitative real-time polymerase chain reaction (qRT-PCR). In this study, the expression profile of 17 candidate genes was assessed in *L. edodes* under treatment at 50 °C for 0, 1, 2, and 3 h, and the expression stability of each reference gene was analyzed by three statistical algorithms, including geNorm, NormFinder, and BestKeeper. Results indicated that the two optimal reference genes for mycelium and fruiting body were *CAC* and *DAHP* as well as *CAC* and *NUP*, respectively. Additionally, *CAC* and *DAHP* were found to be the two most stable reference genes across the mycelium and fruiting body set. Our results will provide a genetic foundation for further research on the metabolism genes of sulfur compounds in *L. edodes*.

## 1. Introduction

*Lentinula edodes* (shiitake mushroom), an edible mushroom well known for its nutritional and pharmacological values as well as the bioconversion of agricultural wastes, ranks only second to *Agaricus bisoporus* in production and cultivation in the word [1,2]. The unique characteristics of dried *L. edodes* mushrooms have been highly prized since ancient times, due to their higher amount of nutrients and greater umami flavor than fresh mushrooms [3,4]. The *L. edodes* mushrooms can be dried by hot air, freezing, vacuum, and microwave. Hot-air drying (HAD) is widely used because of low cost and easy operation as well as obvious improvement of the content of sulfur compounds essential for the unique aroma of *L. edodes* [3,5]. However, the molecular mechanism for the hot-air drying of *L. edodes* remains unclear. Therefore, exploring the transcript changes after this process on the basis of RNA-seq may facilitate the understanding of this mechanism. For the sake of its high sensitivity and good repeatability as well as wide dynamic quantification range [6,7], qRT-PCR (quantitative real-time polymerase chain reaction) is widely used to analyze and verify the expression patterns and levels of genes regulating the metabolism of sulfur compounds after the drying process.

However, the accuracy of qRT-PCR is dramatically affected by various technical factors, such as the quality of RNA, efficiency of RNA reverse transcription, specificity and amplification of primers, and gene expression normalization [8,9,10]. The purpose of normalization is to minimize the effect of nonbiological variation on gene expression. Currently, the application of one or more reference genes, whose expression levels should remain basically unchanged in any experiment conditions, different test tissues, and cells, is the common normalization method in qRT-PCR analysis [11,12,13]. The use of unstable reference genes can generate a negative effect on the result of the relative expression analysis [14]. An increasing number of reports have demonstrated that the expression of any housekeeping genes is stable in specific tissues and under certain experimental conditions [15,16,17,18], suggesting that the screening of proper reference genes for normalization is pivotal to guarantee the accuracy and reliability of results. 

Several studies have been performed on the selection and evaluation of stable reference genes under different conditions, such as different development stages and nutrient conditions as well as abiotic stresses in *Morchella* [19], *Ganoderma lucidum* [20,21,22], *Volvariella volvacea* [23,24], and *L. edodes* [25,26]. Nevertheless, the information about the reference genes used for qRT-PCR normalization of the hot-air-dried *L. deodes* is rather limited. Recently, the RNA-seq between fresh mushroom and mushroom dried by hot air at 50 °C for 3 h was performed in our laboratory (unpublished data). 

In this study, we aimed to detect the reference genes that are stably expressed in the hot-air-dried *L. edodes*. Seventeen candidate reference genes were selected from our in-house *L. edodes* transcriptome data. Three different algorithms (geNorm, NormFinder, and Bestkeeper) were used to evaluate the expression stability of the reference genes after *L. edodes* drying. The reference genes selected in this study could provide a genetic basis for understanding the regulatory mechanism of mushroom drying on sulfurous aroma metabolism in *L. edodes.*

## 2. Results

### 2.1. Selection of Candidate Genes with Stable Expression Using RNA-seq Data

Based on the FPKM value, 10 genes with an absolute log2-fold value of 1 were selected. Meanwhile, another seven housekeeping genes reported in the literature were also tested. All 17 selected genes contained conserved domains and were mapped to protein database (Supplementary Table S1). The full-length sequences of candidate reference genes were obtained from the genome database of *L. edodes* (http://legdb.chenlianfu.com/) and used for the following primer design. The standard curves were generated from 10-fold serial cDNA dilutions by qRT-PCR, and the threshold cycle (Ct) was used to normalize for each standard curve. Amplification efficiency (E) was measured as 10^−1/slope^ − 1 and expressed in percentage. All the primers showed high amplification E values from 91.2% to 109.5%, and high linear correlation coefficients (R^2^) from 0.984 to 1.000. Characteristics of candidate reference genes used in this study are presented in Table 1. The melting curve results revealed the candidate genes as a single peak except for the *Actin* gene (Supplementary Figure S1). *Actin* melting curve showed a smaller shoulder, with a 750 bp secondary PCR product (data not shown). The amplicon size, PCR efficiency, and minimum and maximum threshold cycle (Ct) values were observed across fresh *L. edodes* mycelium. The standard curves (Supplementary Table S2) were used to calculate PCR efficiency.

### 2.2. Expression Profiles of Candidate Reference Genes

**Figure 1:**
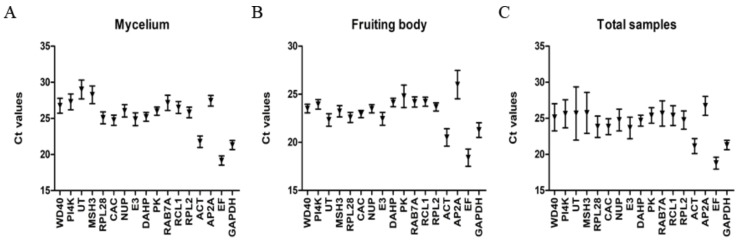
Variation in qRT-PCR values of candidate reference genes.

Data from three technical repetitions of three biological replicates (a total of nine Ct values) were averaged and used for further analysis. The mean Ct values of mycelia were 19.17 for *EF* and 29.01 for *UT*, with the mean value of 12 genes in the range of 25 to 30. The *EF*, *GAPDH*, *ACT*, *CAC*, and *E3* were highly expressed and their Ct values were smaller than 25. Moreover, the top five with the minimum relative variation were *PK*, *DAHP*, *GAPDH*, *EF*, and *CAC* (Figure 1A). While the mean Ct values of fruiting bodies were 18.41 for *EF* and 26.01 for *AP2A*, the remaining 15 genes had a mean Ct value between 20 and 25, and the top five with the smallest relative variation were *CAC*, *DAHP*, *NUP*, *RPL2*, and *PI4K* (Figure 1B). A comparison of set A and B showed that the mean Ct values of all the 17 candidate reference genes were higher in mycelium than in fruiting body. Obviously, the Ct value of most of the candidate reference genes showed a greater relative variation in set C than in set A or set B (Figure 1C).

The mean Ct values of the candidate reference genes are shown on the y-axis, and the 17 candidate reference genes are shown on the X-axis. Error bars represent SD values. Figure 1A shows the mycelium data set, with the mean Ct value of each candidate reference gene being the arithmetic mean value of four different mycelial samples; 1B, the fruiting body data set, with the mean Ct value of each candidate reference gene being the arithmetic mean value of four different fruiting body samples; 1C, the all-sample data set, with the mean Ct value of each candidate reference gene being the arithmetic mean value of eight samples.

### 2.3. geNorm Analysis

The geNorm software calculates the gene expression stability (M) of a reference gene and excludes the gene with the highest M value, allowing ranking of the tested genes according to their expression stability. When the threshold value of M is larger than 1.5, the candidate reference genes are not accepted. To determine the optimal number of reference genes in a special experimental condition, the geNorm software can also be used to calculate the pairwise variation (V) by applying a cutoff value of 0.150.

**Figure 2:**
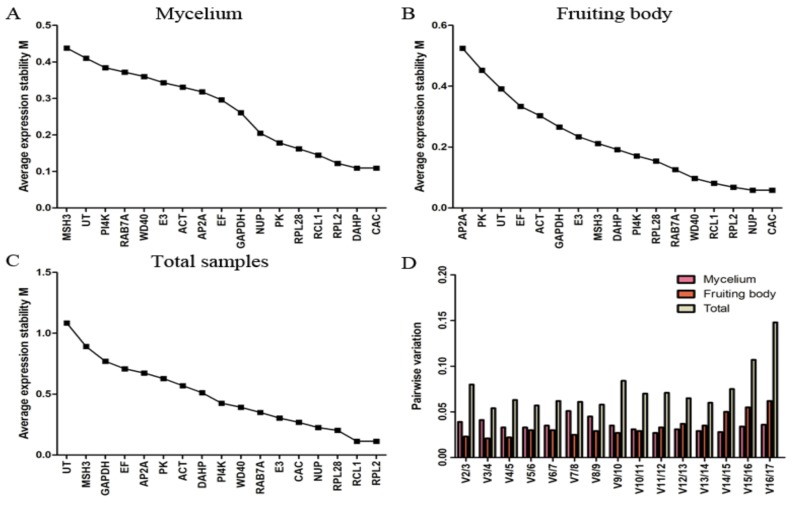
Expression stability and ranking of candidate reference genes determined by geNorm. (**A**) mycelium data set; (**B**) fruiting body data set; (**C**) all-sample data set; (**D**) pairwise variation (Vn/Vn + 1) for the optimal number of reference genes.

Figure 2 shows the expression stability of the candidate genes for fruiting body and mycelium under high-temperature stress over different time periods. For set A, *CAC* and *DAHP* were most stable, while *MSH3* was clearly less consistently expressed (Figure 2A). For set B, *CAC* and *NUP* were most stable, while *AP2A* was the least stable gene (Figure 2B). For the total sample data set, *RPL2* and *RCL1* were indicated as the most stable genes, while *UT* and *MSH3* were indicated as the least stable ones (Figure 2C). It can be seen that *CAC* was the most stable gene in both fruiting body and mycelium, but it was less stable in set C. While *RPL2* and *RCL1* were indicated as the genes with the most stable expression in set C, they were ranked at the third and fourth position in set A and B, respectively. Overall, the M values of all candidate genes were less than 1.5, indicating that all the candidate reference genes could be accepted in the three sample data sets of *L. edodes*.

The evaluation of pairwise variation under each experiment condition is presented in Figure 2D. According to a Vn/Vn+1 cutoff value of 0.150 for pairwise variations, the first n reference genes are sufficient for accurate normalization. In this article, the highest pairwise variation values for any gene pair were 0.051 for set A, 0.062 for set B, and 0.148 for set C, indicating that the optimal number of reference genes was two genes in all three sample sets of *L. edodes*, with *CAC* and *DAHP* for mycelium, *CAC* and *NUP* for fruiting body, and *RPL2* and *RCL1* for the entire data set. 

### 2.4. NormFinder Analysis

NormFinder, which takes into account the intragroup and the intergroup variation, also calculates an M index. Table 2 shows the ranking of the candidate reference genes by NormFinder. For mycelium, *NUP*, *CAC*, *RPL2*, *RPL28*, and *RCL1* were predicted as the top five stable genes with the M values of 0.066, 0.138, 0.142, 0.147, and 0.158, respectively. *MSH3* showed the lowest stability value not only in geNorm but also in NormFinder. For fruiting body, *RCL1*, *MSH3*, *DAHP*, *E3*, and *NUP* were estimated as the most stable genes with the stability values of 0.059, 0.096, 0.100, 0.102, and 0.123, respectively. *AP2A* showed the least stability again. For total sample set, *RCL1*, *RPL2*, *NUP*, *E3*, and *RPL28* were determined as the most stable genes.

### 2.5. BestKeeper Analysis

BestKeeper, a program that can analyze up to 10 reference genes, was used to evaluate the expression stability of the 10 potential reference genes, including all the top five most stable genes determined by both geNorm and NormFinder. The BestKeeper analysis results are shown in Table 3. In the analysis, the two requirements of SD [±Ct] < 1 and SD [±x-fold] < 2 were strictly followed when determining the stability of the selected genes [27], and the gene with the lowest SD was considered as the one with the most stable expression. For mycelium and fruiting body, all the analyzed genes passed this filter, and after calculation of all samples, satisfactory parameters were only obtained for *CAC* and *DAHP*. Another indicator for higher gene expression stability is the r value close to 1 [27], and the minimum r value was 0.891, demonstrating that the correlation coefficients for stability were high for all the 10 genes.

### 2.6. Validation of Reference Genes

**Figure 3:**
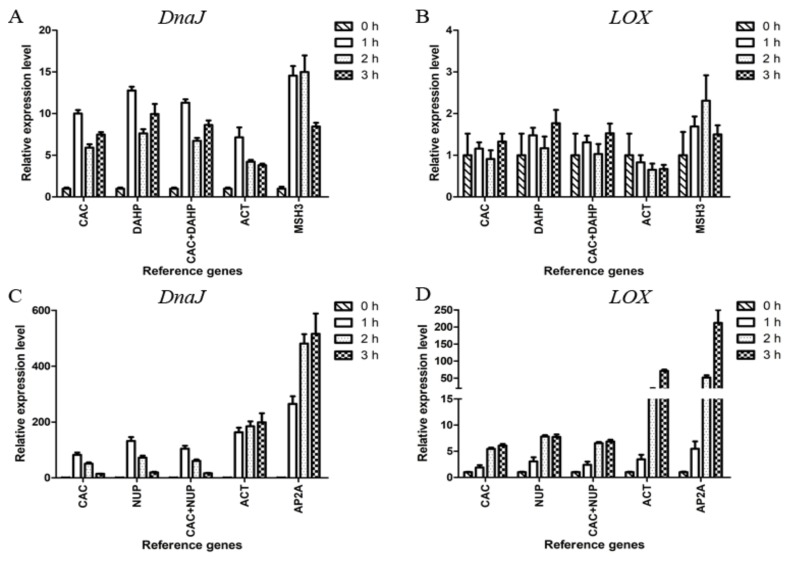
Validation of the selected reference genes. Relative expression of *DnaJ* and *LOX* in mycelium (**A**, **B**) and fruiting body (**C**, **D**). In mycelium, *CAC* and *DAHP* were the most stable candidate reference genes, and *MSH3* was the least stable candidate reference gene. In fruiting body, *CAC* and *NUP* were the most stable candidate reference genes, and *AP2A* was the least stable candidate reference gene. *ACT*, commonly used as endogenous control, was also adopted. Data are shown as mean ± standard deviation (n = 3).

The reliability of the candidate reference genes was evaluated by analyzing the relative expression levels of the heat shock protein 40 kD (DnaJ) and lipoxygenase (LOX) in *L. edodes* under the treatment of 50 ℃ for 0, 1, 2, and 3 h (Figure 3). 

The heat treatment results indicated that *CAC* and *DAHP* were the most stable reference genes, and *MSH3* was the least stable one in mycelium. In Figure 3A, it can be seen that the relative expression levels of *DnaJ* obtained by normalizing against *CAC*, *DAHP*, or *CAC+DAHP* showed obvious differences from those obtained by normalizing against *ACT* or *MSH3*. The relative expression levels of *LOX* showed significant changes only when normalized against *MSH3* at 2 h (Figure 3B).

The above results indicated *CAC* and *NUP* were the most stable reference genes, and *AP2A* was the least stable one in fruiting body. In Figure 3C, the relative expression levels of *DnaJ* showed a similar trend when normalized against *CAC*, *NUP*, or *CAC+NUP* in contrast to an increasing trend from 1 to 3 h when normalized against *ACT* or *AP2A*. In Figure 3D, every group showed an upward trend. In the case of 3 h, the expression levels of *LOX* showed a 6.86-fold upregulation when normalized against the two stable genes *CAC+NUP* in contrast to an overestimated 211.64-fold upregulation when normalized against the least stable gene *AP2A*.

### 2.7. Application of Selected Reference Genes for qRT-PCR Analysis of Heat-Regulated Genes

In order to investigate the effective of reference genes *CAC*, *DAHP*, and *NUP* for qRT-PCR analysis in *L. edodes* after hot-air drying, we determined the expression levels of four heat-regulated genes (Le01Gene04167, Le01Gene02680, Le01Gene11038, and Le01Gene02954) [28] and two flavor genes in the generation of sulfur compounds (Le01Gene02830 and Le01Gene03297) [29]. 

**Figure 4:**
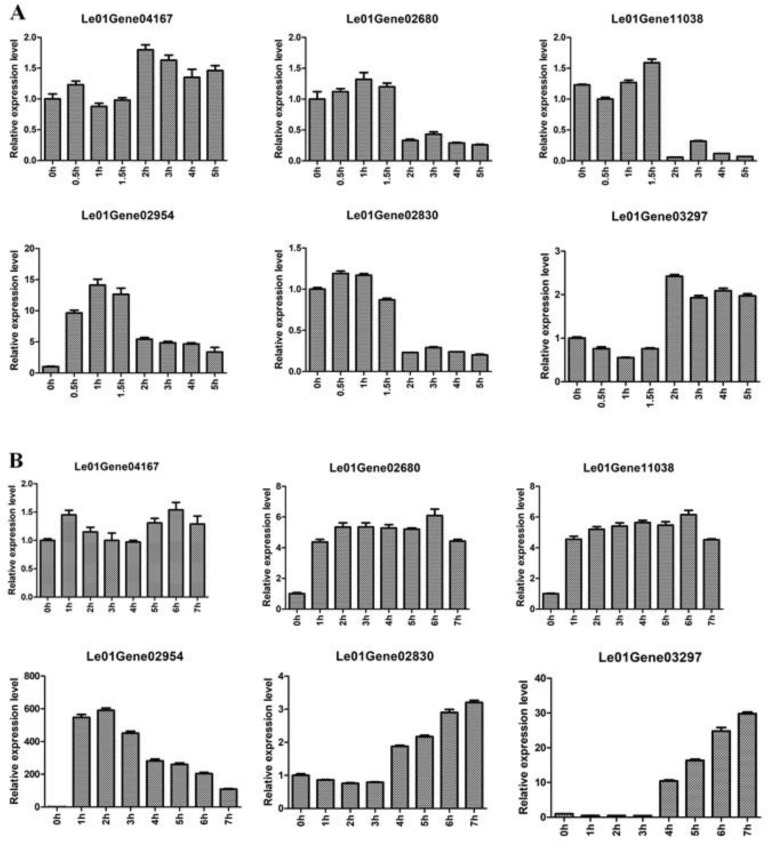
Relative expression of different genes during hot-air drying. Mycelia was treated with 0, 0.5, 1, 1.5, 2, 3, 4, and 5 h (**A**). Fruiting body was treated with 0, 1, 2, 3, 4, 5, 6, and 7 h (**B**).

As shown in Figure 4A, the expression levels of Le01Gene04167 and Le01Gene03297 in mycelia were stable during 5 h of hot-air drying. The expression levels of Le01Gene02680, Le01Gene11038, and Le01Gene02830 had no obvious change at 0.5, 1, and 1.5 h of hot-air drying, whereas the three genes’ expression levels significantly decreased at 2, 3, 4, and 5 h. In addition, the expression levels of Le01Gene02954 encoding the heat shock protein 98 increased during 5 h of hot-air drying. In the fruiting body (Figure 4B), the relative expression of Le01Gene04167 was kept at a similar level during 7 h of hot-air drying. However, the expression levels of three heat-regulated genes Le01Gene02680, Le01Gene11038, and Le01Gene02954 increased during 7 h of hot-air drying. Moreover, the expression levels of the two flavor genes Le01Gene02830 and Le01Gene03297 displayed no obvious change at 1, 2, and 3 h while their expression levels significantly increased at 4, 5, 6, and 7 h.

## 3. Discussion

The stability of internal reference genes is a prerequisite for the reliability of qRT-PCR results. In early reports, several housekeeping genes such as *Actin* and *GAPDH* as well as *TUB* were selected as the internal reference genes because of their functions involved in the basic cytoskeleton of organelles and basic biochemical metabolic processes of organisms [15,16]. In later studies, the expression levels of the housekeeping genes were found unstable under abiotic and biotic stress [26,30], suggesting the importance of reliable reference genes for qRT-PCR analysis. The stability of reference genes was evaluated under different development stages, nutrient conditions, and abiotic stresses in *G. lucidum*, *V. volvacea, L. edodes*, *and Pleurotus ostreatus* [21,24,26,31]. In the present study, we focused on the reference genes for shiitake mushroom in the early stage of the hot-air drying process. As a complex environmental factor, hot-air drying stress was reported to affect the global gene expression in commercial baker’s yeast [32]. When ripe strawberries were heat-treated in an oven (3 h at 45 °C), the expression of key genes controlling cell wall remodeling was found to be changed [33]. In addition, many heat shock proteins have been found in shiitake mushrooms during heat stress [28]. 

In this study, 17 candidate reference genes, including 7 reported in the literature, were obtained from the in-house transcriptome data and analyzed by geNorm, NormFinder, and BestKeeper. Firstly, the amplification specificities of 17 candidate reference genes were evaluated by the melting curves and agarose gel electrophoresis. A single product was observed in the melting curves and gel electrophoresis, and the R^2^ values of all the 17 candidate reference genes were greater than 0.98, indicating a reliable linear relationship between the respective Ct values and the log values of the initial gene copy numbers. The E values met the requirement of 90% to 110%. These results suggested the high specificity and amplification efficiency of the 17 candidate reference genes. 

*Actin* and *GAPDH* as well as *EF* are involved in cytoskeleton biosynthesis and fundamental metabolism of organisms and should be stably expressed in physiological states and cells [34,35]. Nevertheless, *Actin* was ranked in the 11th (0.570) and 10th (0.486) place, and *EF* and *GAPDH* in the 14th (0.708 and 0.686) and 15th (0.770 and 0.946) place by geNorm and NormFinder analyses among the 17 candidate reference genes, respectively, indicating that these three housekeeping genes were relatively less stable after hot-air drying of the shiitake mushroom, which was consistent with several previous reports. Under heat stress and different development stages as well as various nutrient conditions, *Actin* and *GAPDH* as well as *EF* were unstably expressed in *L. edodes* [25,26]. In many plants such as cucumber and potato, *Actin* genes were found to be inconstant under salinity stress [16,36]. For gene expression normalization in rice diurnal/circadian, *Actin* and *GAPDH* as well as *EF* were also unstably expressed [37]. 

According to the results of geNorm and NormFinder, two genes (*RCL1* and *RPL2*) encoding 18S rRNA biogenesis protein RCL1 and ribosomal protein L2 exhibited the highest stability. *RPL4* encoding ribosomal protein L4 was the most stably expressed gene and recommended as the best reference gene for qRT-PCR analysis under different nutrient conditions in *G. lucidum* and *L. edodes* [20,25]. Besides, *RPL4* was considered as the best reference gene in different tissues for *G. lucidum* [20]. As good housekeeping genes with constitutive expression in organism, *RPL13* and *RPL9* encoding the ribosomal protein were selected as the most stable reference genes for gene expression analysis in tested organs from chickens and mice, respectively [38,39]. Nevertheless, no reports are available about the use of the 18S rRNA biogenesis protein RCL1 as the reference gene. This is probably the first report of *RCL1* as the most stable reference gene for gene expression analysis after the shiitake mushroom hot-air drying as determined by geNorm and NormFinder. Meanwhile, the BestKeeper analysis showed the SD values of *RCL1* and *RPL4* between *L. edodes* mycelium and fruiting body were greater than 1.0, demonstrating that both of them were not suitable reference genes for normalization in different development stages.

Bestkeeper analysis showed an SD value of less than 1.0 for *DAHP* and *CAC*, displaying their high stability as the pair of reference genes for normalization under experimental conditions for *L. edodes* mycelium and fruiting body. Similarly, *CAC* encoding clathrin adaptor complex has been proven to be the best reference gene for gene expression normalization in zucchini, coffee, banana, tomato, buckwheat, and *Brassica juncea* studies [13,40,41,42,43,44]. To our best knowledge, this is also the first report of *DAHP* as a reference gene, which encodes DAHP synthetase, an important enzyme in the shikimate pathway [45]. 

For normalization at a different heat treatment time of mycelium, *CAC/DAHP* was the best pair of reference genes as determined by geNorm, while *CAC/NUP* was the most ideal pair of reference genes as determined by NormFinder. As indicated by BestKeeper analysis, both the SD value of *CAC/DAHP* and the r value of *DAHP* were the smallest. Based on the integrated data, we suggest *CAC/DAHP* as the best pair of reference genes for normalization of qRT-PCR data in mycelium.

For normalization of a different heat treatment time of fruiting body, the *CAC/NUP* primer pair showed good performance as determined by geNorm, but with a different ranking position when evaluated by NormFinder, which ranked *CAC/NUP* in the eighth and fifth place, and *RCL1/MSH3* as the top two most stable genes. However, *CAC/NUP* achieved a higher ranking than *RCL1/MSH3* in BestKeeper. Besides, the differences between maximum and minimum Ct values of *CAC*, *NUP*, *RCL1*, and *MSH3* genes were 0.89, 0.97, 1.14, and 1.23, respectively. The overall results indicated that *CAC/NUP* could be a better pair of reference genes than *RCL1/MSH3* for normalization of qRT-PCR data in fruiting body.

The reliability of the proposed reference genes was tested using the two most stable genes, the least stable one, and the widely used *ACT* as reference genes to normalize the relative expression of genes versus high temperature. In this study, Vn/Vn + 1 was V2/V3 < 0.150 (Figure 2D) in three data sets, indicating that two reference genes are enough as a standardized factor. Moreover, the normalization of qRT-PCR data using the best two (*CAC/NUP* in fruiting body and *CAC/DAHP* in mycelium) either individually or jointly resulted in similar and reliable results. To ensure high-quality data, we suggest the use of two reference genes for normalization.

The *DnaJ* and *LOX* genes were tested under the indicated conditions due to their crucial role for survival and flavor, respectively [46,47]. Compared with *AP2A* and *ACT*, the use of *CAC* and *NUP* individually or jointly led to opposite trends of *DnaJ* or large gaps of *LOX* in fruiting body (Figure 3C,D). In mycelium, when normalized with different reference genes, inconsistent results were obtained (Figure 3A, 3B). These results highlight that the use of unsuitable reference genes can compromise the normalization of qRT-PCR data.

Given that all qPCR assays are performed with proper controls, we believe that accurate and reliable qRT-PCR analysis results of *L. edodes* under high temperature can be obtained by using the reference genes reported in this study. Collectively, *CAC/DAHP*, *CAC/NUP*, and *CAC/DAHP* were determined as the primer pairs with stable expression in mycelium set, fruiting body set, and all-sample set, respectively. According to the application results of CAC, DAHP, and NUP, our results indicated that the selected reference genes were able to be used for qRT-PCR analysis in *L*. *edodes* after hot-air drying. This study has laid a genetic foundation for further research on the molecular mechanism of the special shiitake flavor change after hot-air wind drying.

## 4. Materials and Methods

### 4.1. Organisms, Growth Conditions, and Sample Harvest

The *L. edodes* strain WX1 (ACCC 50926) was maintained and cultivated in Mushroom Science and Education Center, Huazhong Agricultural University, Wuhan, China. Samples of fruiting body were harvested at mature stage when its inner membrane started to crack naturally. Then, the fresh fruiting body samples were dried by hot air at 50 °C for 0, 1, 2, and 3 h. Similarly, the mycelial samples of *L. edodes* were incubated at 25 °C for 10 days and then treated as described for fruiting body. All samples were frozen in liquid nitrogen and stored at −80 °C for further use. All samples were collected in three biological repetitions.

### 4.2. RNA Isolation, Quality Control, cDNA Synthesis

The total RNA was extracted from samples using a Spin Column Fungal Total RNA Purification Kit (Sangon Biotechnologies, Shanghai, China). RNA concentration and quantity were determined using a NanoDrop 2000 spectrophotometer (Thermo Scientific, USA), and the RNA samples with only one single peak at 260 nm and the OD260/OD280 ratio between 1.8 and 2.2 were used for further analysis. Then, the integrity of RNA was checked by electrophoresis on 1% agarose gel, and the 28S, 18S, and 5S subunit bands were clear (Supplementary Figure S2). Finally, 20 μL cDNA was prepared using 1 μg of total RNA with the HiScript II Q RT SuperMix for qPCR (+gDNA wiper) kit (Vazyme Biotech, Nanjing, China), according to the manufacturer’s instructions. Before qRT-PCR analysis, the cDNA was diluted five times with double-distilled water and stored at −20 °C.

### 4.3. Selection of the Reference Genes Using RNA-seq Data and Primer Design

Novel candidates were selected from *L. edodes* based on DEG profiles of the two samples (fresh fruiting body samples were dried by hot air at 50 ℃ for 0 and 3 h) from our in-house transcriptome data. Genes that were nondifferentially expressed (false discovery rate <0.01 and an absolute log_2_-fold change) were chosen randomly. These genes were annotated with the genome database of *L. edodes* (http://legdb.chenlianfu.com/) [29] and subsequently used in primer design. Additionally, seven housekeeping genes reported in previous studies were included for analysis [25,26].

The primer design principle was as follows: the size of the target fragment was between 100 and 300 bp, the optimal length of primer was at 20 ± 3 bp, the content of G+C ranged from 40% to 60%, and the primers could cross one intron at least. All primers were synthesized by the Tianyi Huiyuan Biotech Co., Ltd. The primer pairs (Table 1) were designed using Primer Premier 5.0 with an amplification product length ranging from 113 bp to 263 bp. The presence of a single PCR product was verified by 2% (m/w) agarose gel electrophoresis before qRT-PCR. The amplification reaction of each pair of primers was performed in a total volume of 20 μL consisting of 10 μL of 2 × Taq Master Mix (Vazyme Biotech, Nanjing, China), 5 μL of 1.6 μM primer mixture, 3 μL of 1:5 diluted cDNA, and 2 μL of ddH_2_O, under the conditions of an initial denaturation for 5 min at 95 °C, 40 cycles of 30 s at 95 °C, annealing at 50 °C for 30 s, and extension at 72 °C for 20 s, followed by maintaining at 72 °C for 10 min. The size of the primer amplification product was consistent with the target fragment, with a single and clear band present under agarose gel electrophoresis, and such primers were selected for subsequent analysis.

### 4.4. Evaluation of Amplification Efficiency of Candidate Reference Genes

Using fresh *L. edodes* mycelium for comparison, the primer pairs were amplified to detect PCR product using cDNA as the template. The amplification efficiency of candidate genes was calculated referring to the method of Zhao et al. [26]. The initial cDNA was diluted to 10^0^, 10^−1^, 10^−2^, and 10^−3^. Standard curves contained 4 points using 10-fold dilution series. Amplification efficiencies (E) and correlation coefficients (R^2^ values) were checked from standard curves, with each repeated three times. 

### 4.5. qRT-PCR Amplification

qRT-PCR analyses were performed on a CFX Connect Real-Time system (Bio-Rad, USA) with AceQ^®^ qPCR SYBR^®^ Green Master Mix (Vazyme Biotech, Nanjing, China). Each qRT-PCR experiment was repeated with three technical replicates. PCR amplification was performed with 1 μL of 1:5 diluted cDNA, 5 μL of absolute AceQ^®^ qPCR SYBR^®^ Green Master Mix (2×), 2.5 μL of 1.6 μM primer mixture, and 1.5 μL of ddH_2_O. All PCRs were run with an initial denaturation for 5 min at 95 °C, 40 cycles of 30 s at 95 °C, annealing at 60 °C for 30 s, extension at 72 °C for 20 s, and melting at 60–95 °C. The specificity of each qRT-PCR reaction was tested using a melting curve, and the presence of a single PCR product was verified by 2% (*m*/*w*) agarose gel electrophoresis.

### 4.6. Data Processing and Statistical Analysis

The qRT-PCR was performed on cDNA from WX1 fruiting body and mycelium under hot-air treatment and the Ct values were recorded. Three sample data sets (A, B, and C) comprising varying combinations of the eight samples (Supplementary Table S3) were used for geNorm and NormFinder analysis, and the ten relatively stable genes were selected for BestKeeper analysis.

### 4.7. Validation of Reference Genes

To confirm the effectiveness of the selected reference genes for data normalization, the expression levels of heat shock protein 40 (DnaJ) and lipoxygenase (LOX) were examined in eight different samples. The data were normalized using the two most stable genes and the least stable reference gene. The qRT-PCR amplification conditions were the same as those described above. The relative expression levels of the three genes were calculated by the 2^−ΔΔCt^ method.

## Figures and Tables

**Table 1:** Description of the candidate reference genes.

Gene	Symbol	Primer Sequence (5′–3′)	Length (bp)	E (%)	R^2^	Min Ct	Max Ct
WD40 protein	WD40	AGTCCGACAAATCCATCAG	138	97.8	0.990	23.81	33.84
		CAATAACCCTCAGACACCC					
Phosphatidylinositol 4-kinase	PI4K	TATCCGAACGGTTACTGG	198	109.5	0.990	23.81	32.98
		AATTGAGGACGACGCTTT					
Urea transporter	UT	AGAGGGTAATCAGAATGGAA	153	104.9	0.984	26.64	35.96
		TTTGTAGTCGAAGTAGGGTG					
MutS Homolog 3	MSH3	GAACCGAGGAGTAAGATTGT	244	91.2	0.992	24.95	35.33
		TGTTCGTAGCCGAGTGG					
Ribosomal protein L28	RPL28	CAAATGCGTGGATAGCG	223	95.7	0.998	22.22	32.44
		CCAAGCAAGTTCCGATGT					
Clathrin adaptor complexes medium subunit	CAC	CACCTTCCCTTTCCACTG	135	93.6	0.994	22.21	32.70
		TTTGCTCCTTTACCACCA					
Nucleoporin	NUP	AACAGTCTAACTTCGGTGCG	193	99.4	0.998	23.00	32.97
		CCTGTCGTTGCCTCCTCA					
Ubiquitin–protein ligase E3	E3	TAAACGGCGGACAAATGC	263	100.8	0.998	21.22	31.05
		ACGACCTACAGGCGAAAT					
DAHP synthetase	DAHP	CCTTTGTCTGGACCTTCTG	113	97.0	0.999	22.12	32.25
		CGAGCCTTTACTCCTTCAC					
Protein kinase	PK	TCGGATTCTTTACCTACTGG	128	104.3	0.997	22.61	32.18
		CATGGATGGCACTTCACA					
Ras-related protein Rab-7a	RAB7A	ACTCGTTCGCTGTATGCC	171	100.0	0.997	23.03	32.86
		GCTGTCCAGACTCCCTATGA					
18S rRNA biogenesis protein RCL1	RCL1	TGCCGTTCGTGTAAATC	176	97.1	0.987	23.74	33.78
		ACAAAGCAGAGGTGGTAGA					
Ribosomal protein L2	RPL2	AACGAGGACAAGGAAGCC	166	102.2	0.996	23.34	33.10
		CCAGGCAATGTTCTCAGTC					
Actin	ACT	CCCATCTTTCCGTCCACT	242	98.8	0.998	18.22	28.25
		TTCTGACCCATCCCAACC					
Adaptor-related protein complex 2 subunit alpha	AP2A	CGATGAGGATTTGGGAGT	178	101.5	0.998	23.44	33.25
		CAGCCAGGGTGAAGGTAC					
Elongation factor	EF	TTCCCAGGCTGATTGTG	176	97.8	1.000	16.46	26.54
		ATCGGTCCTCGCTCCAT					
Glyceraldehyde 3-phosphate ehydrogenase	GAPDH	CATCCCTTCTTCAACTGG	240	96.8	0.998	17.70	27.89
		AAATCGGTGGAGACAACA					

**Table 2:** Ranking of candidate reference genes according to NormFinder.

Ranking	Mycelium		Fruiting Body		Total	
	Gene	Stability Value	Gene	Stability Value	Gene	Stability Value
1	NUP	0.066	RCL1	0.059	RCL1	0.039
2	CAC	0.138	MSH3	0.096	RPL2	0.058
3	RPL2	0.142	DAHP	0.100	NUP	0.077
4	RPL28	0.147	E3	0.102	E3	0.082
5	RCL1	0.158	NUP	0.123	RPL28	0.123
6	DAHP	0.159	RPL2	0.136	CAC	0.218
7	E3	0.164	WD40	0.140	RAB7A	0.237
8	WD40	0.179	CAC	0.149	WD40	0.332
9	PK	0.202	GAPDH	0.153	PI4K	0.415
10	RAB7A	0.205	RAB7A	0.176	ACT	0.486
11	AP2A	0.239	ACT	0.251	DAHP	0.512
12	PI4K	0.248	RPL28	0.256	PK	0.587
13	ACT	0.278	EF	0.280	AP2A	0.611
14	GAPDH	0.300	PI4K	0.281	EF	0.686
15	EF	0.321	PK	0.555	GAPDH	0.946
16	UT	0.358	UT	0.591	MSH3	1.068
17	MSH3	0.415	AP2A	0.726	UT	1.742
Two best genes	NUP/CAC		RCL1/MSH3		RCL1/RPL2	

**Table 3:** Ranking of candidate reference genes according to BestKeeper.

Ranking	Mycelium				Fruiting Body				Total			
	Gene	SD [±Cq]	SD [±x-fold]	r	Gene	SD [±Cq]	SD [±x-fold]	r	Gene	SD [±Cq]	SD [±x-fold]	r
1	DAHP	0.52	1.43	0.993	CAC	0.28	1.21	1.000	DAHP	0.58	1.49	0.953
2	CAC	0.58	1.50	0.979	WD40	0.28	1.22	0.966	CAC	0.91	1.87	0.995
3	RPL2	0.61	1.52	0.990	RPL2	0.30	1.23	0.992	RPL2	1.07	2.10	0.998
4	RPL28	0.67	1.59	0.967	NUP	0.33	1.26	0.995	RCL1	1.14	2.21	0.997
5	RCL1	0.68	1.60	0.987	RCL1	0.34	1.27	0.996	E3	1.22	2.34	0.984
6	NUP	0.74	1.67	0.993	DAHP	0.35	1.28	0.929	RPL28	1.24	2.37	0.99
7	E3	0.75	1.68	0.938	RAB7A	0.36	1.28	0.934	NUP	1.28	2.43	0.999
8	WD40	0.89	1.85	0.992	RPL28	0.39	1.31	0.891	RAB7A	1.48	2.78	0.995
9	RAB7A	0.90	1.87	0.987	MSH3	0.49	1.41	0.955	WD40	1.62	3.08	0.997
10	MSH3	0.98	1.97	0.966	E3	0.56	1.47	0.955	MSH3	2.52	5.73	0.989

## Supplementary Materials

The Supplementary Materials are available online.
