# Supplementary material for: Selection of Reference Genes for qRT-PCR Analysis in Lentinula edodes after Hot-Air Drying
Source: Molecules. 2018 Dec 31;24(1):136. doi: 10.3390/molecules24010136 (PMC6337709; doi:10.3390/molecules24010136)
Supplement: Supplementary file 1 [file molecules-24-00136-s001.pdf]

Table S1. Predicted function of candidate reference genes and their expression levels in samples by RNA-Seq

Gene ID	Gene name	log_2FC	Pfam annotation	KOG annotation
LE01Gene06296	WD40 protein	-0.0045	Anaphase-promoting complex subunit 4 WD40 domain (pfam12894)	WD40 protein DMR-N9; General function prediction only
LE01Gene00750	Phosphatidylinositol 4-kinae	-0.0038	Phosphatidylinositol 3- and 4-kinase (pfam00454)	Phosphatidylinositol 4-kinase; Intracellular trafficking, secretion, and vesicular transport
LE01Gene06427	Urea transporter	0.0003	Solute carrier families 5 and 6-like, solute binding domain(pfam00474)	Urea transporter; Amino acid transport and metabolism
LE01Gene00643	MSH3	0.0014	MutS domain I (pfam01624), MutS domain II (pfam05188), MutS domain III (pfam05192), MutS domain V (pfam00488)	Mismatch repair MSH3; Replication, recombination and repair

LE01Gene09654	Ribosomal protein L28	0.0015	Ribosomal L28 family (pfam00830)	Mitochondrial/chloroplast ribosomal protein L28; Translation, ribosomal structure and biogenesis
LE01Gene04230	Clathrin adaptor complexes medium subunit	0.0037	C-terminal domain of adaptor protein (AP) complexes medium mu subunits and its homologs (MHD) (pfam00928),Clathrin adaptor complex small chain (pfam01217), Domain of unknown function (DUF4670) (pfam15709)	Medium subunit of clathrin adaptor complex; Intracellular trafficking, secretion, and vesicular transport
LE01Gene01221	Nucleoporin	0.0044	Nucleoporin FG repeated region (pfam15967)	Nuclear pore complex, Nup98 component (sc Nup145/Nup100/Nup116); Intracellular trafficking, secretion, and vesicular transport
LE01Gene06776	E3 ubiquitin-protein ligase	0.0066	Ring finger domain (pfam13639)	FOG: Predicted E3 ubiquitin ligase; Posttranslational modification, protein turnover, chaperones

LE01Gene11481	DAHP synthetase	0.0072	DAHP synthetase I family (pfam00793)	No
LE01Gene02300	Protein kinase	0.0074	Protein kinase domain (pfam00069)	Ca2+/calmodulin-dependent protein kinase kinase beta and related serine/threonine protein kinases; Signal transduction mechanisms
LE01Gene13383	Ras protein	0.0097	P-loop containing Nucleoside Triphosphate Hydrolases (pfam08477),Ras family (pfam00071)	Ras-related GTPase;<br>General function prediction only
LE01Gene00881	RNA cyclase	-0.1850	18S rRNA biogenesis protein RCL1 (pfam01137)	RNA 3′-terminal phosphate cyclase；<br>RNA processing and modification
LE01Gene00960	Ribosomal Proteins L2	0.0777	Ribosomal Proteins L2, C-terminal domain (pfam03947), Ribosomal Proteins L2, RNA binding domain (pfam00181)	Mitochondrial/chloroplast ribosomal proteinL2; Translation, ribosomal structure and biogenesis

LE01Gene01050	Actin	0.1999	Actin (pfam00022)	Actin and related proteins; Cytoskeleton
LE01Gene03178	AP-2 complex subunit	-0.2878	Adaptin N terminal region (pfam01602), Alpha adaptin AP2, C-terminal domain (pfam02296), Adaptin C-terminal domain (pfam02883)	Vesicle coat complex AP-2, alpha subunit; Intracellular trafficking, secretion, and vesicular transport
LE01Gene03252	Elongation factor	-0.0369	Elongation factor Tu GTP binding domain (pfam00009), Elongation factor Tu C-terminal domain (pfam03143), Elongation factor Tu domain 2 (pfam03144)	Translation elongation factor EF-1 alpha/Tu; Translation, ribosomal structure and biogenesis
LE01Gene07114	Glyceraldehyde-3-phosphatedehydrogenase	0.2932	Glyceraldehyde 3-phosphate dehydrogenase, C-terminal domain (pfam02800), Glyceraldehyde 3-phosphate dehydrogenase, NAD binding domain (pfam00044)	Glyceraldehyde 3-phosphate dehydrogenase; Carbohydrate transport and metabolism

Table S2. Slopes of qRT-PCR curves for reference genes

Gene	Equation of standard curves	Slope
WD40	y = -3.375 x + 43.992	-3.375
PI4K	y = -3.113 x + 42.607	-3.113
UT	y = -3.209 x + 46.151	-3.209
MSH3	y = -3.553 x + 46.204	-3.553
RPL28	y = -3.430 x + 42.745	-3.430
CAC	y = -3.485 x + 42.919	-3.485
NUP	y = -3.337 x + 42.961	-3.337
E3	y = -3.303 x + 41.006	-3.303
DAHP	y = -3.396 x + 42.455	-3.396
PK	y = -3.223 x + 41.967	-3.223
RAB7A	y = -3.321x + 42.913	-3.321
RCL1	y = -3.393 x + 44.072	-3.393
RPL2	y = -3.269 x + 42.878	-3.269
ACT	y = -3.350 x + 38.166	-3.350
AP2A	y = -3.287 x + 43.108	-3.287
EF	y = -3.375 x + 36.635	-3.375
GAPDH	y = -3.401 x + 37.930	-3.401

Table S3. Eight samples of *L. edodes* involved in this study and the three different combinations of sample sets

No.	Materials	Notes
1	50℃-0h	Mycelium of strain WX1
2	50℃-1h	
3	50℃-2h	
4	50℃-3h	
5	50℃-0h	Fruiting body of strain WX1
6	50℃-1h	
7	50℃-2h	
8	50℃-3h	

The three different combinations of sample sets:

Item	Set	Samples
A	Different heat time of mycelium	Samples of 1,2,3 and 4
B	Different heat time of fruiting body	Samples of 5,6,7 and 8
C	Total samples	Samples of 1,2,3,4,5,6,7 and 8

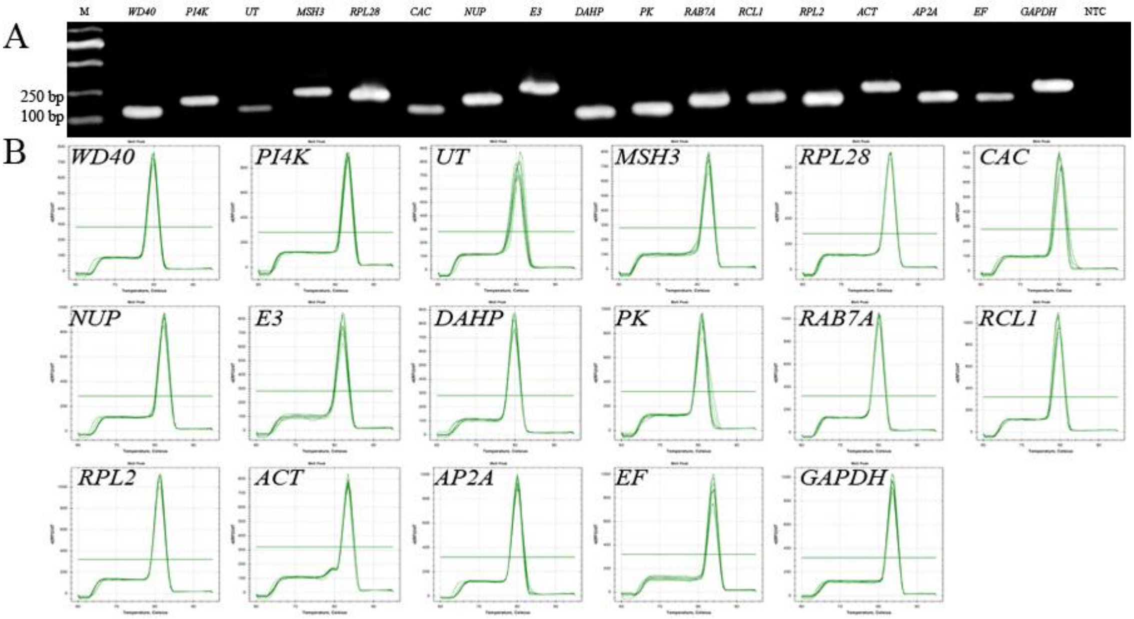

Figure S1. The amplification length and specificity of candidate reference genes.

A: Amplified fragments of candidate reference genes shown by agarose gel electrophoresis with ethidium bromide staining. B: Melting curves generated by qRT-PCR. M: BM2000 DNA marker; NTC: No template control.

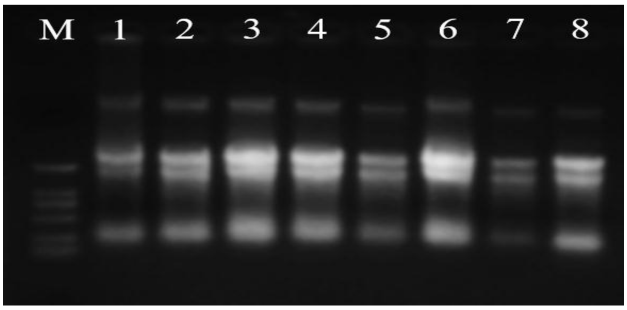

Figure S2. Agarose gel electrophoresis of RNA samples.

M：BM2000 DNA marker.1-4: *Lentinula esodes* fruiting body dried by hot-air at 50 ℃ for 0, 1, 2 and 3 hours, respectively. 5-8: *Lentinula esodes* mycelium dried by hot-air at 50 ℃ for 0, 1, 2 and 3 hours, respectively.
